# Increased prime edit rates in *KCNQ2* and *SCN1A* via single nicking all-in-one plasmids

**DOI:** 10.1186/s12915-023-01646-7

**Published:** 2023-07-13

**Authors:** N. Dirkx, Wout J. Weuring, E. De Vriendt, N. Smal, J. van de Vondervoort, Ruben van ’t Slot, M. Koetsier, N. Zonnekein, Tim De Pooter, S. Weckhuysen, B. P. C. Koeleman

**Affiliations:** 1 Applied & Translational Neurogenomics Group, VIB Center for Molecular Neurology, VIB, Antwerp, Belgium; 2 Department of Biomedical Sciences, University of Antwerp, Antwerp, Belgium; 3 Department of Genetics, University Medical Center Utrecht, Utrecht, 3584 CX The Netherlands; 4 Neuromics Support Facility, VIB Center for Molecular Neurology, VIB, Antwerp, Belgium; 5 Translational Neurosciences, Faculty of Medicine and Health Science, University of Antwerp, Antwerp, Belgium; 6 Department of Neurology, Antwerp University Hospital, Antwerp, Belgium

**Keywords:** Prime editing, EIEE, CRISPR, SCN1A, KCNQ2, Developmental and epileptic encephalopathy, EF-1alfa, Human-induced pluripotent stem cells, Gene editing, Monogenic diseases

## Abstract

**Background:**

Prime editing (PE) is the most recent gene editing technology able to introduce targeted alterations to the genome, including single base pair changes, small insertions, and deletions. Several improvements to the PE machinery have been made in the past few years, and these have been tested in a range of model systems including immortalized cell lines, stem cells, and animal models. While double nicking RNA (dncRNA) PE systems PE3 and PE5 currently show the highest editing rates, they come with reduced accuracy as undesired indels or SNVs arise at edited loci. Here, we aimed to improve single ncRNA (sncRNA) systems PE2 and PE4max by generating novel all-in-one (pAIO) plasmids driven by an EF-1α promoter, which is especially suitable for human-induced pluripotent stem cell (hiPSC) models.

**Results:**

pAIO-EF1α-PE2 and pAIO-EF1α-PE4max were used to edit the voltage gated potassium channel gene KCNQ2 and voltage gated sodium channel gene SCN1A. Two clinically relevant mutations were corrected using pAIO-EF1α-PE2 including the homozygous truncating SCN1A R612* variant in HEK293T cells and the heterozygous gain-of-function KCNQ2 R201C variant in patient-derived hiPSC. We show that sncRNA PE yielded detectable editing rates in hiPSC ranging between 6.4% and 9.8%, which was further increased to 41% after a GFP-based fluorescence-activated cell sorting (FACS) cell sorting step. Furthermore, we show that selecting the high GFP expressing population improved editing efficiencies up to 3.2-fold compared to the low GFP expressing population, demonstrating that not only delivery but also the number of copies of the PE enzyme and/or pegRNA per cell are important for efficient editing. Edit rates were not improved when an additional silent protospacer-adjacent motif (PAM)-removing alteration was introduced in hiPSC at the target locus. Finally, there were no genome-wide off-target effects using pAIO-EF1α-PE2 and no off-target editing activity near the edit locus highlighting the accuracy of snc prime editors.

**Conclusion:**

Taken together, our study shows an improved efficacy of EF-1α driven sncRNA pAIO-PE plasmids in hiPSC reaching high editing rates, especially after FACS sorting. Optimizing these sncRNA PE systems is of high value when considering future therapeutic in vivo use, where accuracy will be extremely important.

**Supplementary Information:**

The online version contains supplementary material available at 10.1186/s12915-023-01646-7.

## Background

CRISPR/Cas9 was first used for genome editing of eukaryotic cells in 2013 [1], after which it rapidly evolved into a genome editing tool that has revolutionized the field of biology as a whole. Not only did it lead to an unprecedented increase in our capacity to study gene function and the effects of genetic variation on health and disease but it also brought us closer to the implementation of true precision medicine: the ability to apply treatment strategies specifically targeting the cause of disease; the underlying gene mutation. Prime editing (PE), the most recent gene editing tool in the field, uses a Cas9 nickase (Cas9n) protein fused to a reverse transcriptase (RT) and a PE guide RNA (pegRNA) consisting of a guide, a primer binding site, and RT template containing the desired edit [2]. PE is the first gene edit system that can introduce all four possible base pair (bp) transversions, small insertions, and deletions. The first-generation prime editors consist of PE2 (pegRNA + Cas9n-RT) which introduces a single stranded break (nick) at the target locus and PE3 (pegRNA + Cas9n-RT + additional nicking RNA (ncRNA)) which generates a second nick via ncRNA activity. PE3 enhanced editing efficiencies by 1.5- to 4.2-fold compared to PE2 in immortalized cell lines, but at the cost of off-target editing near the intended edit locus such as insertions, deletions (indels), or single nucleotide variants (SNVs). The second-generation PE systems have a human codon-optimized RT, a further optimized Cas9n (PEmax), and chemically modified pegRNAs that increase stability [3]. PE2 and PE3 systems that have these enhancements can furthermore be co-expressed with MutL homolog 1 (MLH1dn), which is a DNA mismatch repair (MMR)-inhibiting protein, and are then named PE4max and PE5max, respectively.

Since PE systems that use Cas9n depend on a NGG protospacer-adjacent motif (PAM), a short DNA sequence of 2–6 base pairs immediately following the DNA sequence that is targeted for editing, the edit rates can be further increased if the PAM is disturbed after the intended edit is introduced. This was shown recently by repeated prime editing rounds that included PAM removal by introducing a silent mutation in this region, leading to an increase of 20% in overall edit activity compared to repeated editing without a PAM-removing mutation [4]. When the PAM is removed, Cas9n can no longer bind to the target DNA, thereby acting as a protection from ongoing PE activity that potentially could reverse the initial correction and could increase risk of off-target editing. Another improvement to PE has been the modification of pegRNAs at the 3′ terminus which increases stability and prevent degradation [5].

In recent years, several studies showed that double ncRNA (dncRNA) plasmid based PE systems (PE3 and PE5) can also edit more complex in vitro model systems such as human embryonic stem cells (ESC) [6], hiPSC [7], and liver or intestinal organoid cells [8]. Editing of human ESC was achieved via installing a doxycycline inducible PE enzyme gene in the genome followed by electroporation of pegRNA and ncRNA, leading up to 40% edit efficiency [6]. ESC that did not express the PE gene endogenously resulted in an edit efficiency below 1%, which was attributed to difficulties in delivering the PE machinery via lipid-based transfection. In liver and intestinal organoid cell suspensions, electroporation of PE3 showed edit efficiencies up to 50% after sorting for green fluorescent protein (GFP) that was co-delivered from a separate plasmid [8]. Corrected cells were selected and expanded and found to be functionally rescued of their disease phenotype. In all the examples, the PE3 system however introduced undesired indels near the edit locus. In addition, the PE machinery was delivered via several separate (up to four) plasmids, which can result in the uneven distribution of the editor enzyme, pegRNA, ncRNA, and/or an additional selection plasmid such as a fluorescent marker.

Recently, an all-in-one plasmid (pAIO) was published that contains the complete PE3 system (PEA1) thus facilitating transfection of stem cells with all the necessary constructs at once [9]. The pAIO-PE3 plasmid, combined with a puromycin selection, showed a significant increase in edit efficiency compared to the regular PE3 system in mouse stem embryonic stem cells, with edit efficiencies ranging between 1.7% and 85%. In line with other dncRNA PE systems, pAIO-PE3 activity however also resulted in a high rate of indel/SNV generation occurring in up to 90% of the clones.

Since dncRNA PE systems are accompanied by undesired indels/SNVs, improved versions of single ncRNA (sncRNA) PE systems (PE2 and PE4) would be interesting alternatives. Crucial for hiPSC editing using a plasmid-based approach is the use of the correct promoter. Most studies use the original plasmids containing a cytomegalovirus (CMV) promoter, which is known for its weaker expression in stem cells [10]. To our knowledge, only the study that generated PEA1 has changed the original CMV promoter to a chicken β-actin hybrid (CBh) promoter; however, no comparison with the original CMV promoter was performed to validate the effect of the promoter in the editing efficiency. The elongation factor 1 alfa (EF-1α) represents a promoter that has been reported to drive strong expression in hiPSCs and is stable during hiPSC differentiation [10–12]. For these reasons, we generated pAIO-EF1α-PE2 and pAIO-EF1α-PE4max plasmids, driven by the EF-1α promoter and show that our pAIO plasmids can efficiently correct disease related variants in human embryonic kidney 293 T (HEK293T) cells and a patient-derived-hiPSC line.

## Results

### Introduction of single nucleotide variants in HEK293T using multi-plasmid PE systems

**Fig. 1:**
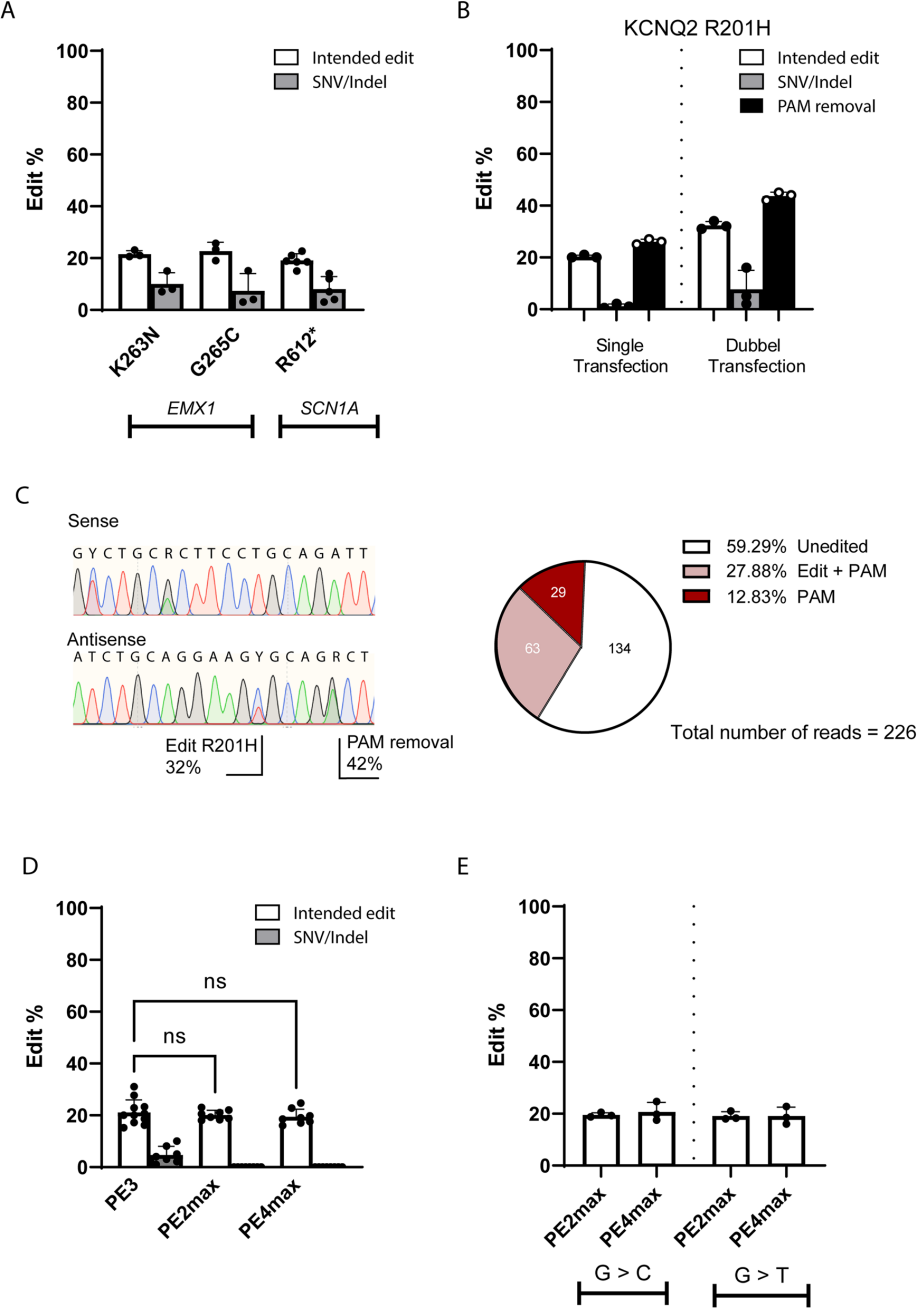
Introduction of DEE mutations using PE3, PE2max, and PE4max in HEK293T cells. White bars represent editing of the target of interest, grey bars represent presence of unintended SNVs/Indels at the edit locus, and black bars represent editing of the PAM site. **A** Validation of PE3 editing using pegRNAs targeting *EMX1* (introduction of K263N and G265C) and *SCN1A* (introduction of R612*). **B** Editing efficiency using PE3 with a pegRNA that introduces the *KCNQ2* R201H mutation together with an additional silent PAM-removing mutation following a single or double transfection. **C** Left: Sanger Sequencing results of R201H + PAM-removing mutation after two transfection rounds. Right: WES read count pie chart of R201H + PAM-removing mutation. **D** Comparison of PE3, PE2max, and PE4max editing efficiency using three different pegRNAs. **E** Comparison of G > C versus G > T conversion efficiency in *SCN1A* at position c.1863–1865, using PE2max and PE4max. The target region and designs of R612* and R201H + PAM pegRNAs can be found in Additional file 1: Fig. S9. Bar plots show the mean edit percentages ± SD; replicates are presented as individual data points (see individual data values in Additional file 2: Table S1)

As a proof of principle, we replicated an experiment from the original PE publication in HEK293T cells using PE3 to introduce mutations in *EMX1* (K263N and G265C) [2]. We also introduced a mutation in a new target, *SCN1A* R612*. The *SCN1A* R612* truncating variant is a pathogenic variant that was previously described in an individual with the prototype developmental and epileptic encephalopathy (DEE) Dravet Syndrome and gave rise to a severe seizure phenotype that resulted in acute encephalopathy leading to death [13]. Sequencing results of the edited pools showed similar edit efficiency for EMX1 K362N, EMX1 G265C, and SCN1A R612* (21.6% ± 1.3, 22.7% ± 3.4 and 19.1% ± 2.6, respectively) (Fig. 1A). As expected for the dncRNA PE3 system, all loci showed unintended Indels/SNVs (Fig. 1A).

Next, the *KCNQ2* R201H mutation was introduced together with an additional silent PAM-removing mutation that could disturb pegRNA cutting once it is installed. *KCNQ2* R201H is a pathogenic Gain of Function variant that leads to a severe neonatal-onset encephalopathy with prominent startle-like myoclonus, infantile onset epilepsy, and a burst-suppression electroencephalography pattern [14]. For a graphical overview on PAM-removal and the four possible outcome scenarios, see Additional file 1: Fig. S1. After the first round of transfection with PE3, both the edit and the silent PAM-removing mutation were confirmed (Fig. 1B). Interestingly, Sanger Sequencing indicated that the edit levels of silent PAM-removing mutation exceeded that of R201H (Fig. 1C left). A second transfection was performed on the same cell pool of cells and further increased the edit levels for both R201H and the PAM-removing mutation (Fig. 1B). We performed whole exome sequencing to validate R201H edit efficiencies and to determine whether the exceeded rate of PAM removal was the result of a biased polymerase chain reaction (PCR) amplification and Sanger sequencing. Whole exome sequencing (WES) analysis confirmed that approximately 28% of the reads carry both R201H and the PAM-removal alteration (Fig. 1C right and Additional file 1: Fig. S2). Interestingly, another 12% carried only the silent PAM-removing mutation, whereas there were no reads detected with just R201H These findings highlight that Sanger sequencing gives comparable results as next generation sequencing (NGS) for PE3 experiments for R201H and PAM removal in HEK293T cells. Furthermore, introduction of an addition silent PAM-removing alteration can be favored over the intended edit (scenario #2 and #4 according to Additional file 1: Fig. S1).

Next, we compared the edit efficiency of the second generation, PE2max and PE4max systems to PE3 for three pegRNAs in two different loci. All three editing systems are approximately equally effective; however, unintended Indels/SNVs were detected for PE3, highlighting the challenge of dncRNA PE (Fig. 1D, Additional file 1: Fig. S3). Due to the PE4max ability to inhibit MMR, it was previously shown to have higher edit efficiencies compared to PE2max. Interestingly, we did not observe this for our edited loci.

Finally, because C to G conversions can be more efficiently introduced as they evade MMR [3], we also validated if changing a NGG motif in *SCN1A* at position c.1863–1865 (NM_001165963.4) to NCG (p.L622V) would be more efficient compared to NTG (p.L622M). No increased efficiency was observed for L622V compared to L622M using PE2max or PE4max (Fig. 1E). Both observations (comparison of PE4max to PE2max and the C to G conversion) can be explained by the MMR deficiency of HEK293T cells, which limit the MMR-inhibition effect of PE4max and the advantage of specific base changes.

### Generation of all-in-one EF-1α driven PE systems

**Fig. 2:**
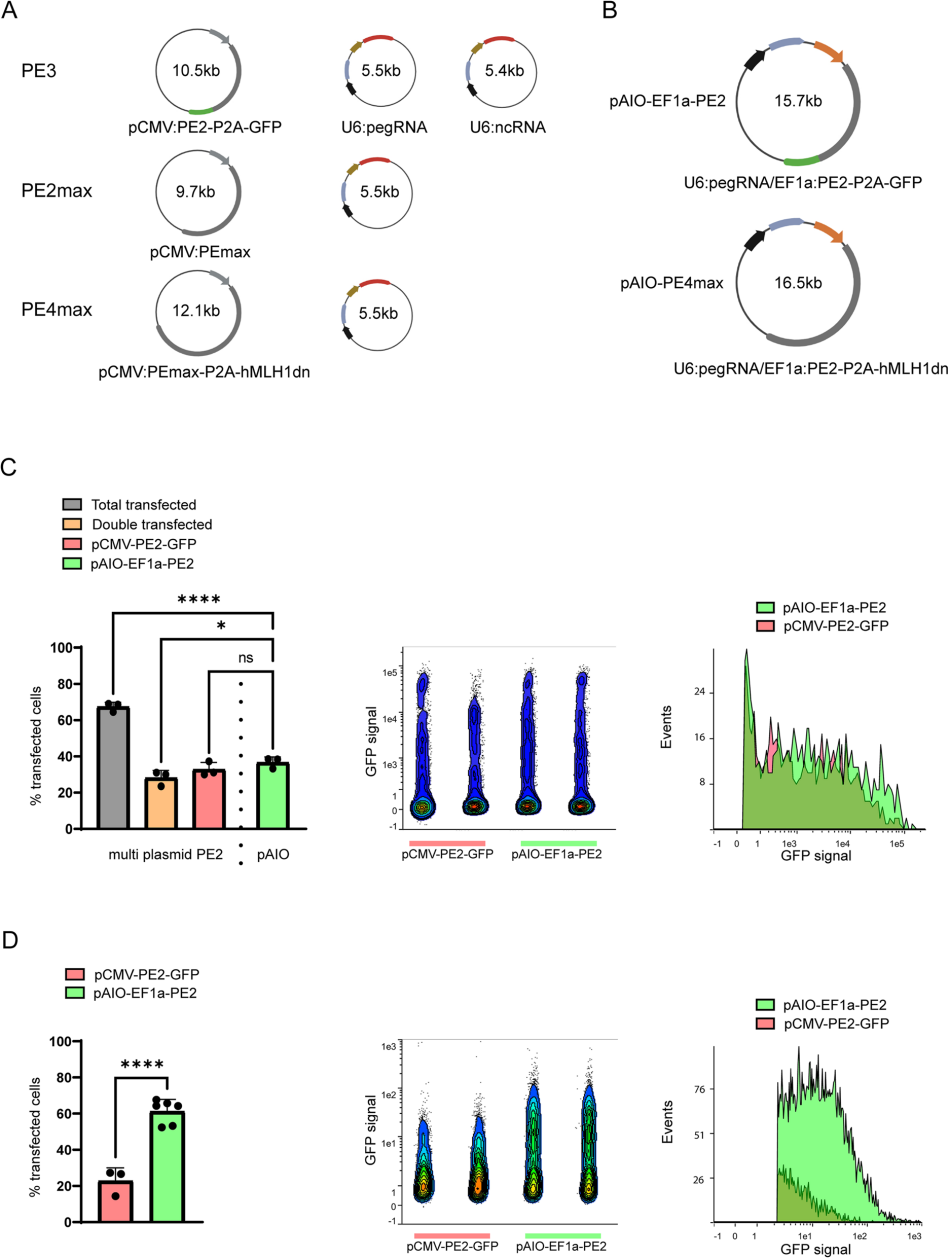
Generation and delivery validation of all-in-one EF-1α driven sncRNA PE plasmids. **A**, **B** Overview of PE systems used in this study. **A** Regularly used CMV-driven multi plasmid PE systems. **B** Overview of the two EF-1α driven pAIO plasmids generated for this study. Plasmids are abbreviated to pAIO-PE2-GFP and pAIO-PE4max from here on. Grey arrow: CMV promoter; grey rectangle: PE system; green rectangle: GFP; black arrow: U6 promoter; blue rectangle: pegRNA; brown arrow: hsyn promoter; red rectangle: mCherry; orange arrow: EF-1α promoter. **C**, **D** Comparison of pCMV-PE2-GFP delivery versus pAIO-PE2-GFP delivery in **C** HEK293T cells and **D** hiPSC, using FACS. **C** (Left) Quantitative representation of the fluorescent fractions when transfected with the full multi plasmid PE2 system, pCMV-PE2-GFP and U6-pegRNA-mCherry (total, double positive and GFP only), compared to pAIO-PE2-GFP in HEK293T. **C**, **D** (Middle) GFP expression profile of **C** the transfected HEK293T or **D** nucleofected hiPSC pools. **C**, **D** (Right) Zoom in on the GFP positive cells of a representative sample set. **D** (Right) Quantitative representation of the fluorescent fractions when transfected with pCMV-PE2-GFP compared to pAIO-PE2-GFP in hiPSC. Bar plots show the mean edit percentages ± SD; replicates are presented as individual data points (see individual data values in Additional file 2: Table S1)

As an improvement to current CMV-driven PE systems that require several plasmids (Fig. 2A), we generated two novel all-in-one (pAIO) EF-1α-driven PE systems on a lentiviral backbone using molecular gene cloning. The EF-1α promoter is known for its high activity and stability in hiPSCs over time and is also described as one of the most stable promoters during hiPSC differentiation [11, 12]. Because ncRNA-based PE systems typically lead to additional unintended SNVs and indels, we combined the PE2-P2A-GFP enzyme [2] with a pegRNA to yield pAIO-EF1α-PE2 and one of the most recent editing enzymes PEmax-MLH1dn [3] with a pegRNA to generate pAIO-EF1α-PE4max (Fig. 2B).

Because the size of a plasmid influences transfection efficiency, we validated the delivery efficiency of our pAIO-EF1α-PE2 plasmid which is 1.5 to 3 times larger than a single independent PE plasmid from multi-plasmid PE systems. We transfected HEK293T cells with pAIO-EF1α-PE2 and compared it to multi-plasmid PE2 transfections using the original pCMV-PE2-P2A-GFP and a mCherry-tagged pegRNA. The number of GFP positive cells transfected with pAIO-EF1α-PE2 was comparable to pCMV-PE2-P2A-GFP (32.8% ± 3.9 versus 36.8 ± 2.9, *P* > 0.05) (Fig. 2C, left) as well as the signal intensity (Fig. 2C, middle and right). However, for the multi plasmid PE2 system, a significantly smaller subset of the transfected cells showed full delivery (double positive) compared to the total amount of transfected cells (28.2% ± 4.1 versus 67.4% ± 2.5, *p* < 0.0001) and the compared to our pAIO-EF1α-PE2 system (28.2% ± 4.1 versus 36.7% ± 2.9, *P* < 0.05) (Fig. 2C, left).

Next, we validated the delivery of pAIO-EF1α-PE2 and pCMV-PE2-P2A-GFP in hiPSCs 48 h after nucleofection. Similar to the results in HEK293T cells, both constructs showed GFP expression in hiPSCs (Fig. 2D). Interestingly, the percentage of GFP positive cells was significantly higher in the hiPSCs when using our EF-1α driven pAIO-EF1α-PE2 compared to pCMV-PE2-P2A-GFP (61.24% ± 6.68 vs 22.77% ± 7.28, *P* < 0.001) (Fig. 2D, left). Furthermore, GFP expression intensity was higher for our pAIO-EF1α-PE2 construct compared to the pCMV-PE2-P2A-GFP construct (Fig. 2D, middle and right), indicating a clear benefit of Ef-1α promoter over the CMV promoter in hiPSC lines.

Together, these data show the effective delivery of pAIO-EF1α-PE2 in HEK293T cells and hiPSC and highlight the importance of an all-in-one plasmid combined with an optimal promoter for high expression in hiPSC.

### Removal of homozygous pathogenic SCN1A R612* variant using pAIO systems in HEK293T

**Fig. 3:**
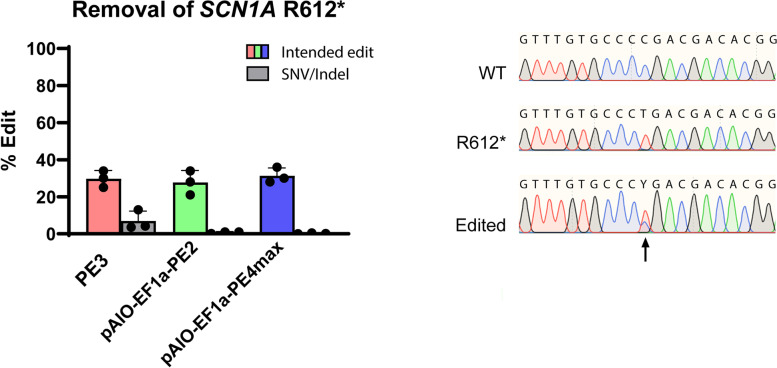
Correction of *SCN1A* R612* in HEK293T^R612*/R612*^. (Left) Comparison of the editing efficiency in correcting the SCN1A R612* mutation using dncRNA PE3 versus sncRNA pAIO-PE2-GFP and pAIO-PE4max. (Right) Sanger Sequencing results of HEK293T^wt/wt^, HEK293T^R612*/R612*^ and treated HEK293T.^R612*/R612*^ cells using pAIO-PE2-GFP. Black arrow indicated the SCN1A R612 location. The target region and design of R612* and *612R pegRNAs can be found in Additional file 1: Fig. S9. Bar plots show the mean edit percentages ± SD; replicates are presented as individual data points (see individual data values in Additional file 2: Table S1)

Using PE3, we generated a homozygous R612* HEK293T cell line via single cell sorting and clonal expansion. To validate if pAIO plasmids are functional for the correction of this mutation, HEK293T^R612*/R612*^ cells were transfected with pAIO-EF1α-PE2 and pAIO-EF1α-PE4max and compared to PE3. All three PE systems pAIO-EF1α-PE2, pAIO-EF1α-PE4max, and PE3 repaired the *SCN1A* R612* mutation on average for 27.7%, 31.3%, and 29.7% of the cell population, respectively, validating that pAIO plasmids are functional (Fig. 3). No undesired SNVs or indels were detected in pAIO-transfected cells, in contrast to those transfected with PE3 (Fig. 3).

To determine if lentiviral vectors (LVV) and integrase-deficient lentiviral vectors (IDLVV) can be produced using the pAIO-EF1α-PE2 plasmid, the third-generation lentiviral vector packaging system was used. First, we generated GFP-LVV and GFP-IDLVV which show stable, and declining GFP expression over time, respectively, validating the transient nature of integrase-deficient lentiviral vectors in dividing cells. In contrast to GFP-IDLVV, pAIO-PE2-IDLVV GFP levels were below detection limit. In line with absence of GFP expression, only marginal correction of *SCN1A* R612* in HEK293T^R612*/R612*^ (< 1%) was observed using pAIO-PE2-IDLVV, highlighting that further optimization for transient delivery of large lentiviral cargo is necessary (Additional file 1: Fig. S4).

### Removal of heterozygous pathogenic KCNQ2 R201C variant in patient-derived hiPSC

**Fig. 4:**
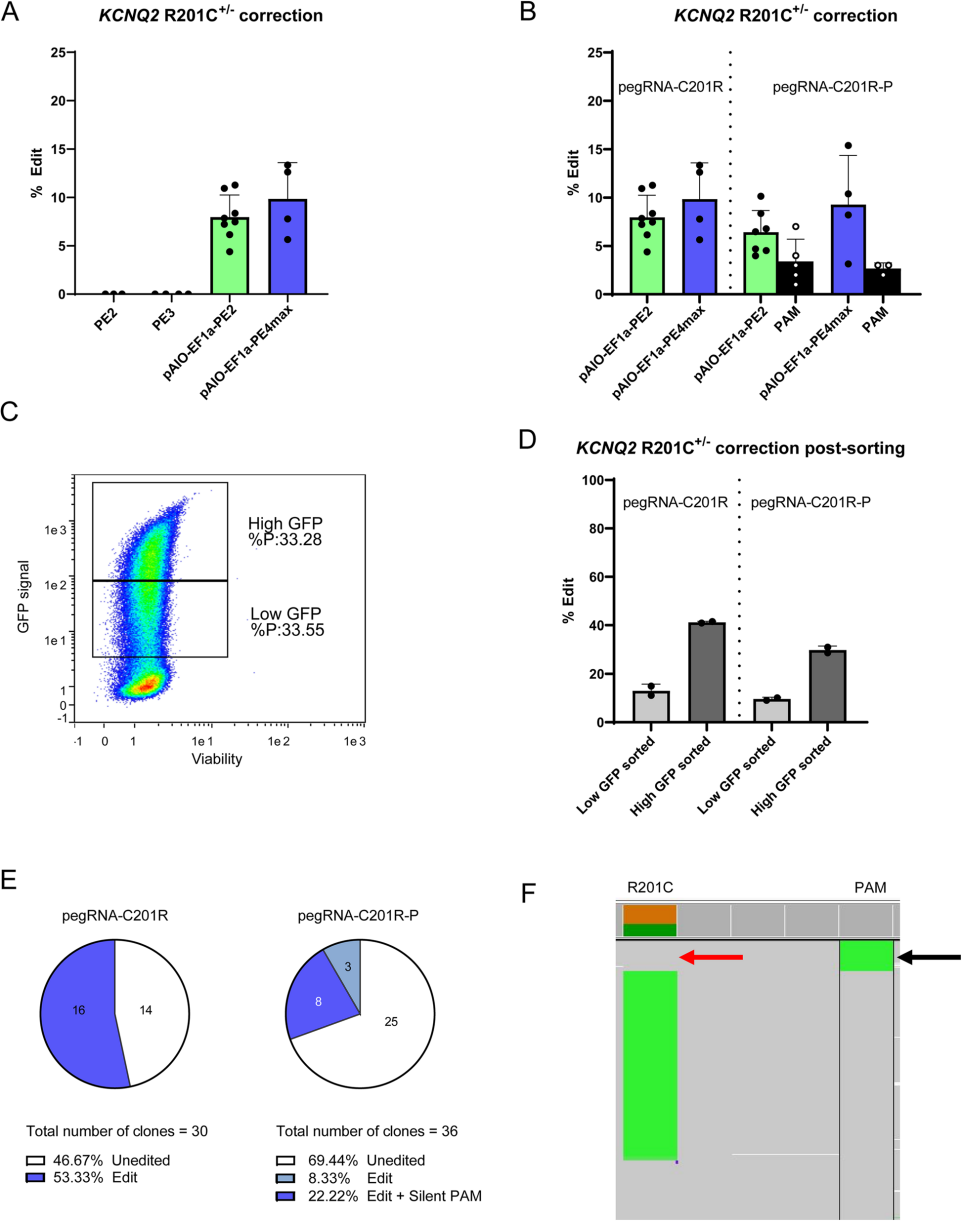
Patient-derived hiPSC editing using pAIO-PE2-GFP and pAIO-PE4max. **A** Comparison of the editing efficiency when correcting the heterozygous KCNQ2 R201C mutation using CMV-PE2-GFP and CMV-PE3-GFP versus pAIO-PE2-GFP and pAIO-PE4max. **B** Comparison of the editing efficiency when correcting heterozygous KCNQ2 R201C mutation using pegRNA-C201R (only correction KCNQ2 R201C) versus pegRNA-C201R-P (correction KCNQ2 R201C + introduction PAM-removal mutation) using pAIO-PE2-GFP and pAIO-PE4max. **C** FACS showing the two GFP-positive fractions that were separated when sorting the GFP populations. **D** Editing efficiency of pAIO-PE2-GFP after sorting on the high and low GFP expressing fractions using pegRNA-C201R and pegRNA-C201R-P. **E** Number of clones that carry the R201C correction using the (left) pegRNA-C201R and (right) pegRNA-C201R-P. The target region and design of pegRNA-C201R and pegRNA-C201R-P can be found in Additional file 1: Fig. S9. **F** Miseq data of pAIO-PE2-GFP- pegRNA-C201R-P high GFP population showing that the PAM-removal mutation (black arrow) is only present in reads that do not carry the KCNQ2-R201C mutation (red arrow). Bar plots show the mean edit percentages ± SD, replicates are presented as individual data points (see individual data values in Additional file 2: Table S1)

To validate the efficiency and accuracy of pAIO-EF1α-PE2 and pAIO-EF1α-PE4max in a more advanced model system, we tested if these plasmids could correct the heterozygous pathogenic variant *KCNQ2* R201C in a patient-derived hiPSC line using a pegRNA that only recognizes the mutant allele (pegRNA-C201R) and compared them to the standard PE2 or PE3 systems. Nucleofected hiPSCs were harvested after 72 h and based on Sanger sequencing and NGS, we could not detect any edits nor indels for the PE2 and PE3 systems (Fig. 4A), which is in line with a previous study using PE3 in hiPSC [6]. On the other hand, nucleofection of hiPSC with pAIO-EF1α-PE2 and pAIO-EF1α-PE4max showed an average edit efficiency of 8.1% and 9.8%, respectively (Fig. 4A). More importantly, for both pAIO-EF1α-PE2 and pAIO-EF1α-PE4max, no unintended SNVs or indels were detected.

Because the timepoint of harvest after transfection of hiPSCs differs in the literature (ranging from 48 to 96 h), we then performed an experiment using only pAIO-EF1α-PE4max to validate edit efficiency at 48 h vs 72 h vs 96 h. No significant differences in edit efficiency were observed between the different time points (Additional file 1: Fig. S5). To find out if introducing an additional PAM-removing mutation could further increase editing in hiPSC, an additional pegRNA was designed (pegRNA-C201R-P). In contrast to our expectation based on previous findings [4, 6], disturbing the PAM did not increase efficiency of PE at this locus and even seem to lower the editing efficiency (Fig. 4B). Interestingly, while the silent PAM removing mutation negatively affected edit efficiency, it was virtually absent when assessed using Sanger Sequencing (Additional file 1: Fig. S5). However, validation with NGS showed the presence of the PAM removing mutation in 3.4% and 2.7% of the reads for pAIO-EF1α-PE2 and pAIO-EF1α-PE4max, respectively, highlighting the limited resolution of sanger to pick up low levels of mosaicism.

We next aimed to improve edit efficiency through addition of a Fluorescence-activated Cell Sorting (FACS) step. Since pAIO-EF1α-PE2 and pAIO-EF1α-PE4max showed similar efficiencies in hiPSC for our target, pAIO-EF1α-PE2 was chosen for FACS sorting due to the benefit of a GFP tag and the absence of the MLH1dn MMR inhibitor which has a potential risk of introducing unwanted DNA mismatches. Furthermore, we sought to investigate if the GFP signal intensity is a marker for edit efficiency. hiPSCs were sorted 48 h after nucleofection with pAIO-EF1α-PE2-pegRNA-C201R or pAIO-EF1α-PE2-pegRNA-C201R-P. During sorting, the GFP positive population was divided in two to separate the top half of the GFP signal (high GFP) from the bottom half of the GFP signal (low GFP signal) (Fig. 4C). Edit levels showed a remarkably high efficiency in the high GFP population up to 41% and a smaller increase for the low GFP population up to 13% for pegRNA-C201R (Fig. 4D). These increased efficiencies were replicated, but to a lower extent, when using pegRNA-C201R-P, reaching 29% and 10% for the high and low GFP population, respectively (Fig. 4D). These data show that FACS sorting on the highest GFP signal can significantly increase the edit efficiency by fivefold, suggesting that more PE2 exposure increases editing, and that for this target a PAM-removing mutation was not beneficial.

Next, using the high GFP populations, we generated clonal lines to validate the edit efficiencies. For the pegRNA-C201R condition, 16 out of 30 clones (51%) presented a correction (Fig. 4E, left), whereas for the pegRNA-C201R-P condition only 11 out of 36 clones (31%) showed a correction (Fig. 4E, right). Eight out of 11 (73%) corrected clones carried the PAM-removal mutation. NGS analysis of the pegRNA-C201R-P condition furthermore showed that the PAM-removal mutation only occurred in corrected reads and was not present in any of the reads still carrying the R201C mutation (Fig. 4F). Interestingly, this data is in complete contrast to our findings in HEK293T that revealed a favor towards the PAM mutation over the introduction of the KCNQ2-R201H mutation and a small percentage of cells with solely the PAM-removing mutation but not the KCNQ2-R201H mutation alone.

Taken together, we show here for the first time that a PE2 plasmid-based system can efficiently introduce mutations in hiPSC without any selection step. We also show that pAIO-EF1α-PE4max is an equally effective system compared the pAIO-EF1α-PE2 for correcting the KCNQ2-R201C mutation. Using GFP-based FACS sorting, we could further increase the edit percentage of the pAIO-EF1α-PE2 nucleofected population by fivefold. Finally, the introduction of an additional silent-PAM removing mutation was not beneficial for this locus.

### Quality control of hiPSC lines and Whole genome sequencing of corrected R201C clones

We validated 91 clones for one of the most frequently recurring copy number variants (CNV) during hiPSC culturing, the chr20q11.21 duplication [15, 16]. This duplication decreases apoptosis which could be favorable during subcloning hiPSC lines. Using Multiplex Amplicon Quantification (MAQ), we verified that none of our clones carried the duplication (Additional file 1: Fig. S6). Next, we validated in a subset of corrected clones the expression of pluripotency markers Oct4, Nanog, and Sox2 (Additional file 1: Fig. S7). Two corrected clones of this subset were selected to perform Whole genome sequencing (WGS) to verify the absence of PE mediated off-target effects. WGS analysis of the two corrected hiPSC clones and the parental hiPSC line shows that no alterations were made within 250 bp of all loci with homology to the pegRNA with a mismatch threshold of 4 bp. We did identify 146 and 139 novel variants respectively in the whole genome of the two corrected hiPSC clones (data not shown). These variants followed an expected distribution of intergenic:intronic:exonic variants (47:51:2) and were not located in a region homolog to the pegRNA, and the majority of variants found (58%) were C:G > A:T substitutions, indicating they are probably the result of accumulation of mutations during cell culture conditions through oxidative stress mechanisms.

## Discussion

In this study, we developed and tested two new all-in-one (AIO) plasmids containing the full PE2 and PE4max PE system. These sncRNA PE systems showed efficient introduction or correction of pathogenic variants in the DEE genes *SCN1A* and *KCNQ2* in both HEK293T and hiPSC without off-target events.

Our experiments in HEK293T cells demonstrated efficient introduction of six different mutations in three different genes (*SCN1A*, *KCNQ2*, and *EMX1*) with edit efficiencies ranging between 19 and 31%, using PE3. However, we observed a high yield of unintended alterations in the editing region for PE3, in line with previous reports. This downside was not observed using the second-generation PE systems, PE2max, and PE4max, which showed equal editing efficiency compared to PE3 based on three pegRNAs for two different loci. Since PE4max inhibits MMR, it was previously found to yield higher edit efficiencies compared to PE2max [3]. Interestingly, we did not observe this for our edited loci in HEK293T cells. Because C to G conversions can be more efficiently introduced as they evade MMR [3], we also validated if changing a NGG motif in *SCN1A* at position c.1863–1865 (NM_001165963.4) to NCG (p.L622V) would be more efficient compared to NTG (p.L622M). No increased efficiency was observed for L622V compared to L622M using PE2max or PE4max. Both observations can be explained by the MMR deficiency of HEK293T cell, which limits the MMR-inhibition effect of PE4max and the advantage of specific base changes.

The few studies that have shown efficient plasmid-based PE editing in stem cells all use the PE3 system with an additional enrichment step [6, 8, 9, 17]. In line with the original publication on the discovery of PE3, all the studies reported unintended indels and SNV near the target locus. The multi-plasmid PE2 system, although more accurate, has shown lower editing efficiencies than PE3, making it less attractive [7]. However, as accuracy is an important factor in the generation of hiPSC-derived disease models, in vivo editing, and CRISPR therapeutics, we aimed at improving the editing rates of plasmid based PE2 systems. In this study, we show that the optimized PE2 systems, pAIO-EF1α-PE2 and pAIO-EF1α-PE4max, are highly efficient as well as accurate. We increased the functionality of the plasmid by changing the CMV promoter to the Ef-1α promoter, which has been shown to be a much stronger promoter in stem cells [11, 12] and neurons [18]. In addition, it is one of the most stable promoters in hiPSC and during in vitro hiPSC differentiation [19]. We show that the pAIO-EF1α-PE2 plasmid, although much larger in size than the individual size of three or more plasmids used for other PE systems, can be easily delivered to HEK293T and hiPSC. As expected, based on promoter activity, pAIO-EF1α-PE2 showed a significant increase in the number of cells expressing GFP compared to pCMV-PE2-P2A-GFP in hiPSC, as well as a higher general GFP expression level. This highlights the importance of choosing the right promoter when applying plasmid-based gene-edit systems and may have been a contributing factor to the low success rate of previous studies using CMV-PE2/PE3 in hiPSCs.

In our study, we show that pAIO-PE plasmids are effective tools for correcting the heterozygous *KCNQ2* R201C mutation in a patient-derived hiPSC line. pAIO-EF1α-PE2 and pAIO-EF1α-PE4max showed a remarkable correction of 8.1% and 9.8%, respectively. This level of efficiency for sncRNA PE systems in hiPSC has not been shown before and proves that plasmid based sncRNA PE2 can efficiently edit hiPSC lines without a selection procedure. To further increase edit efficiency, we sorted the pAIO-EF1α-PE2 nucleofected hiPSC population using FACS. We show that selection of the highest GFP expressing cell population leads to 3- and fivefold increased edit efficiency as compared to the lower GFP expressing cell population and unsorted cells, respectively (41% vs 13% and 8%). Subcloning of sorted hiPSC resulted in a correction of 53% of the R201C clones. This illustrates that more copies of PE enzyme and/or pegRNA per cell improves edit efficiency. Indeed, both the addition of a hairpin structure to the pegRNA that reduced the rate of degradation [5] and controlling the expression window of PE via a doxycyclin-induced expression system led to increased edit efficiency [6], suggesting that increased PE:pegRNA interactions are key for successful PE experiments. In this context, it is important to note that the use of an EF-1α promotor instead of a CMV promotor significantly increased expression of our construct in hiPSC, hence contributing to the increased editing efficiency. This impacted the efficient generation of subcloned hiPSC lines by significantly decreasing the workload for colony picking and clone validation.

Removal of the PAM has been shown to increase edit efficiency when applying PE3 to the *APP* gene. A protective Alzheimer disease variant, A673T, was introduced after repeated PE rounds in 68.9% of the pool of cells, while 49.2% was edited if PAM-removal was not introduced [4]. It was hypothesized that by disturbing the NGG motif, Cas9n could no longer interact with the target site DNA, and after an initial round of editing resulted in PAM removal, the locus was protected from subsequent unintended edit rounds. We tested the PAM removing R201H-P pegRNA that introduces the R201H mutation in HEK293T cells and observed an increased level of PAM-removal editing compared to editing of R201H. We observed that a small but significant portion of the cells carried only the PAM-removing mutation, while no cells carried solely the R201H edit. In contrast, removal of the R201C mutation in patient-derived hiPSC revealed a small number of clones with only the C201R correction, but no clones with PAM removal alone. Contrary to what has been suggested previously, we observed increased edit rates for the regular pegRNA when comparing a pegRNA with PAM removal. The reason why a PAM-removing edit is favored over R201H editing in HEK293T, and the contrary is seen in hiPSC, is currently unclear but several hypotheses can be made. First, HEK293T cells have homozygous wildtype *KCNQ2* alleles, whereas the patient-derived hiPSC carry a heterozygous R201C allele which, since the pegRNAs are allele-specific, can result in an altered number of PE:gDNA interactions. Secondly, the pegRNA design could play a role. While the PAM removing edit involves a C to T alteration in both cases, R201H editing involves G > A whereas C201R is a T to C conversion. Finally, both pegRNAs target the sense allele, but C201R-P is one base longer on the 3′ site. Although this is not expected to introduce large differences, its influence cannot be excluded. In general, although PAM removal is claimed to be beneficial for editing outcome, our observations at the *KCNQ2* R201 locus show this is not always the case. In relation to our HEK293T experiments, cells that have a disturbed PAM motif but not the intended edit, cannot be targeted anymore as Cas9n is unable to cut the target DNA, and thus PAM-removal might act contraproductive.

Although PE3 and PE5 were not tested in this study, our EF-1α driven pAIO plasmids could be used for this purpose via introducing an extra ncRNA together with the pAIO-EF1α-PE2 or pAIO-EF1α-PE4max, in case dncRNA-dependent off targets are acceptable.

Finally, using WGS in two edited clonal lines, we could not find any genome-wide off-target effects at loci with homology to the pegRNA, confirming prior evidence of the safety of the PE2 technology. As an outline for the future, we believe that our system might be further optimized with smaller Cas variants and cloning on a smaller plasmid backbone. We here used the pLV backbone, which carries genes needed for lentiviral production. These however can be taken out if the aim is to use pDNA, downsizing the system with at least 5 kb. Since we show that > 15 kb pDNA with the PE machinery can be easily delivered to hiPSC with a nucleofection efficiency of over 60%, smaller pDNA can further improve delivery and thereby edit efficiency. Finally, improved lentiviral vector production for large cargos aimed at transient expression, for example via non-reverse transcriptase or integrase-deficiency, would enable efficient delivery to neuronal cell models and can further advance prime editing in vivo.

## Conclusions

Over the last few years, double ncRNA based PE3 has shown successful editing results in more complex model systems such as stem cells and stem cell-derived cultures. In our study, we prove that the safer single ncRNA-based PE system that has been reported to have low editing rates can now edit HEK293T cells and iPSCs with high efficiency when using EF-1α driven all-in-one plasmids. Due to minimal chance of on target SNVs or indels when using the single ncRNA PE systems, we believe that our optimized single ncRNA systems are of added value for in vivo editing or CRISPR therapeutics where accuracy is most important.

## Methods

### PegRNA cloning for PE3, PE2max, and PE4max

pegRNAs and ncRNAs were cloned into *AAV-U6-sgRNA-hSyn-mCherry* (Addgene #87,916). The pegRNAs and ncRNAs were designed using PegFinder [20]. One hundred micrometers of single-stranded DNA oligos were ordered via IDT in MilliQ. Oligos were annealed by mixing 10 µL of each oligo in 20 μL of 2X annealing buffer (20 mM Tris pH 7.5, 100 mM NaCl and 20 mM EDTA) and ran in the following PCR program; 5 min on 95 °C, start on 95 °C and – 2 °C/s for 4 s, start on 85 °C and − 0.1 °C/s for 599 s and incubation on 4 °C. The AAV-U6-mCherry plasmid was linearized by SapI following manufacturer’s instructions. Ligation of the oligos was performed by T4 DNA Ligase (NEB M0202L) following manufacturer’s protocol and transformed using *TOP10* competent cells. Plasmids were purified using Qiagen mini or midiprep kits and verified by Sanger Sequencing. For oligo sequences, see Additional file 1: Fig. S8. For a graphical overview of all pegRNA used in this study, see Additional file 1: Fig. S9.

### Generation of pLV-EF-1α Prime Edit plasmids

pLV-EF1a-PE2-P2A-GFP was generated by cloning the original pCMV-PE2-P2A-GFP insert (Addgene #132776) into an EF-1α driven lentiviral backbone by Signagen Inc (from here on called pLV-PE2). DNA oligos were designed for introduction of new restriction sites in pLV-PE2. Single-stranded DNA oligos were annealed following previous described protocol. Digestion of the backbone was performed by ClaI (NEB #R0197L) according to manufacturer’s instructions. Ligation of the annealed oligos in the linear backbone was performed by T4 DNA Ligase. pLV-EF1a-PEmax-P2A-hMLH1-dn was generated from the pLV-PE backbone. The pCMV-PE4max backbone was linearized by AgeI (NEB #R3552L), and MluI restriction sites were introduced via molecular gene cloning according to above methods with the exception that *Stbl3* and not *TOP10* competent cells were used. The pCMV-PE4max with MluI restriction site and the pLV-PE plasmid were both digested by MluI (NEB #R0198L) and NotI (NEB #R0189L). PEmax-P2A-hMLH1-dn insert was ligated in the pLV-EF1alpha backbone and transformed in *Stbl3* competent cells. pLV-EF1a-PE2-P2A-GFP (#184445) and pLV-EF1a-PEmax-P2A-hMLH1-dn (#184444) are available on Addgene after publication. Plasmids were verified using Sanger Sequencing. For oligo sequences see Additional file 1: Fig. S8

### pegRNA cloning and generation of pAIO-EF1α-PE2 and pAIO-EF1α-PE4max

PegRNAs were ordered as 500 ng gBlock (double stranded DNA fragments) via IDT and resuspended in 10μL MilliQ prior to cloning in the dual PaqCI (NEB #R0745L) site of pLV-EF1a-PE2-P2A-GFP (from here on called pAIO-EF1α-PE2) or pLV-EF1a-PEmax-P2A-hMLH1-dn (from here on called pAIO-EF1α-PE4max). Digested plasmids and gBlock DNA fragments were purified by Wizard SV Gel and PCR clean-up system (Promega), annealed according to the manufacturer’s instructions, and transformed in *Stbl3* competent cells. pAIO-EF1α-PE2:KCNQ2-C201R (#185060) and pAIO-EF1α-PE2:KCNQ2-C201R_pp (#185061) are available on Addgene after publication. Plasmids were verified using Sanger Sequencing. For oligo sequences, see Additional file 1: Fig. S8. For a graphical overview of all pegRNA used in this study, see Additional file 1: Fig. S9.

### Prime editing transfections in HEK293T cells

Twenty-four hours prior to transfection, HEK293T cells were seeded to yield approximately 50% confluency at day 1 in 6 wells plates. For PE3, three different plasmids were transfected simultaneously in HEK293T cells; 1 μg of pCMV-PE2-P2A-GFP, 0.5 μg of pegRNA plasmid, and 0.5 μg of nicking RNA plasmid were combined and transfected with lipofectamine according to manufacturer instructions. For PE2max (Addgene #174820) and PE4max (Addgene #174828), 1.4 μg editor plasmid and 0.6 μg of pegRNA plasmid was used. Genomic DNA extraction was performed 72 h after transfection using DNeasy Blood & Tissue Kit from Qiagen. In the case of a second transfection experiment, approximately 20% of the cells remained in culture after splitting and transfected when confluency reached 50% again. The remaining cells were used for gDNA extraction and genotyping. For generation of HEK293T^R612*/R612*^, transfected cells were single cell sorted based on GFP and mCherry co-expression and expanded. Genotyping followed after 2 weeks and a successfully edited clone was selected. For genotyping, the edit locus was PCR-amplified using TaqGold Polymerase, and the PCR product was purified by Wizard SV Gel and PCR clean-up kit followed by Sanger Sequencing.

### Whole exome sequencing on edited HEK293T

Whole exome sequencing was performed on DNA from HEK293T. Methods and the script used are listed at https://github.com/UMCUGenetics/DxNextflowWES and the IGV alignment is added as Additional file 1: Fig. S2.

### Lentiviral vector generation

Lentiviral vectors (LVV) were produced following the third-generation packaging plasmid system in HEK293T cells. The following plasmids were used in a 4:2:1:1 (μg) ratio: transfer plasmid (pAIO, or pLenti-GFP), pMDLg/pRRe (or psPAX2-D64 for integrase deficient lentivirus, IDLVV), pRSV-rev, pMD2.g. pDNA was mixed in 200 µL OptiMEM and combined with 24 µL PEI (1 mg/mL). The DNA:PEI complexes were added to the cells after 20 min incubation at room temperature, and medium was replaced at approximately 18 h. LVV or IDLVV were harvested using the Lenti-X-concentrator from Takara 72 h after transfection according to manufacturer’s protocol. The titer of harvested lentivirus was established using RT-qPCR (Takara Bio, Cat. no. 631235). A representative calibration plot and LV tittering of pAIO-PE2-IDLVV is added as Additional file 1: Fig. S4.

### hiPSC maintenance

The hiPSC line harboring the R201C *KCNQ2* variant was obtained by reprogramming patient PBMCs by the Radboudumc Stem Cell Technology Center (SCTC). The hiPSCs were maintained in StemFlex medium (Thermo Fisher Scientific, A3349401) on Geltrex (Thermo fisher Scientific, A1413302) coated plates at 37 °C and 5% CO_2_. Cells were passaged with ReleSR (Stemcell Technologies, 05872) when they reached 70–80% confluency. The hiPSC line was tested for pluripotency markers (Nanog, Sox2 and Oct4) using qPCR, and showed no abnormalities on CNV analysis.

### Prime editing nucleofection of hiPSC

hiPSC were dissociated using Accutase (Sigma, A6964). Cell pellets of 1E6 cells were resuspended with 20 μl supplemented nucleofector solution of the P3 Primary Cell 4D-nucleofector® X Kit S (Lonza, V4XP-3032) and 2 μg plasmid (1 μg/μl), and nucleofected with the 4D-Nucleofector Core and X Unit (Lonza), with program CB-150. 10 min after nucleofection, cells were plated in StemFlex media supplemented with 10 μM Y-27632 rho kinase inhibitor (Tocris, 1254). Genomic DNA extraction was performed 72 h after transfection, unless stated otherwise, using Macherey–Nagel NucleoSpin DNA mini kit (Macherey–Nagel, 740952) according to manufacturer’s protocol. The edit locus was PCR amplified using Titanium® Taq DNA Polymerase (Takara, 639208) followed by Sanger Sequencing.

### Next generation sequencing of target region to validate editing efficiency and off targets in hiPSC

Off targets were validated using Illumina miseq. PCR amplicons of the target region were generated as described above and prepared for sequencing using the Nextera XT DNA Library Preparation Kit (Illumina, San Diego, CA, USA) and subsequently deep sequenced on a MiSeq instrument (Illumina) using 2 × 250 bp cycles. Miseq results were analyzed using IGV.

Editing efficiency was validated using either Sanger Sequencing (as described above), Illumina miseq (as described above), or long-read Flongle (Oxford Nanopore Technologies (ONT)) sequencing. For ONT, PCR amplicons were generated using tagged primers for barcoding (see Additional file 1: Fig. S8). Barcoding was done using the LongAmp Taq 2X Master Mix (M0287L, New England Biolabs) and PCR Barcoding Expansion 1–96 kit (EXP-PBC096, Oxford Nanopore Technologies) as specified in ONT protocol. Libraries were prepared using the Ligation Sequencing Kit (SQK-LSK110, Oxford Nanopore Technologies). Samples were quality controlled using Qubit (double-stranded DNA [dsDNA] high sensitivity; Thermo Fisher Scientific) and Fragment Analyzer (Agilent Technologies; using a DNF-473 Standard Sensitivity NGS 1-6000 bp kit). All amplicons were pooled and run on a R9.4.1 Flongle Flow Cell (FLO-FLG001 Oxford Nanopore Technologies). Flongle Flow Cell had a minimum of 50 sequenceable pores at the start, and loading was approximately 20 fmol followed by 24 h of sequencing.

The basecalling of the Nanopore data was performed using the Guppy basecaller version v6.0.6. Analysis was performed using a pipeline integrated in genomecomb [21] Reads were aligned to the hg38 genome reference [22] using minimap2[23] and the resulting sam file sorted and converted to bam using samtools [24]. Results were analyzed using IGV.

### Flow cytometry analysis of HEK293t and hiPSC and fluorescence-activated cell sorting (FACS) of hiPSC

Forty-eight hours after nucleofection or transfection, hiPSCs and HEK293t cells were dissociated using Accutase or trypsin, respectively. The pellet was resuspended in 1 ml FACS buffer (PBS with 2% fetal bovine serum), with 1000X Viability Dye eFluor® 780 (Thermo Fisher Sci., 65–0865), and incubated for 20 min on ice. After incubation, 10 ml FACS buffer was added, and cells were centrifuged for 3 min at 300 g to wash the cells. The cell pellet was resuspended in 2 ml FACS buffer and ran through a 30-µm filter. GFP + signal was measured using the MACSQuant® Analyser, and data was analyzed using FlowLogic. Cells for sorting were added to the MACSQuant® sorting cartridge (Miltenyi Biotec). Gating hierarchies were constructed using MACSQuant Tyto software before sorting. Cell debris, doublets, and dead cells were gated out. GFP + signal was divided in two (highest 50% and lowest 50% of the GFP signal). After sorting cells of the lower GFP signal, the positively sorted cells were collected from the cartridge and seeded in StemFlex media supplemented with 10 μM Y-27632 rho kinase inhibitor, the negatively sorted cells were reloaded, and the sorting gate was changed to a higher GFP + signal.

### Subcloning of prime edited hiPSC line

Subcloning of the edited hiPSC lines to generate isogenic clones, was performed by seeding 500 single hiPSCs in one well of a 6 well plate coated with Geltrex, in StemFlex media supplemented with CloneR™ (stem cell technologies, 05888). Three hours after seeding, cells were placed in the Incuctye® Live Cell Analysis System and imaged every 3 h for 7 to 10 days to track if colonies originated from 1 single cell. Cells were kept in CloneR supplement media for 4 days, whereafter the CloneR supplement was removed. Between day 7 and day 10, colonies originating from one single cell were picked and placed in an individual well of a 24 well plated coated with Geltrex and StemFlex media. Clonal lines were split into two 12 wells, one for expansion and one for DNA isolation, to check the absence or presence of the R201C mutation.

### Determination of edit efficiency and off-target activity

Sanger files (.ab1) were uploaded in EditR for determination of edit efficiency and unintended SNVs or indels. Sanger files for Fig. 1D is added as Additional file 1: Fig. S3.

### Whole genome sequencing analysis on edited hiPSC

DNA was extracted from two sorted clones and the naïve parental hiPSC line. Samples were submitted to BGI Denmark for WGS with standard sequencing coverage of 30X/90 Gb data. Reads were aligned to human genome build hg38 using BWA. Single-nucleotide variants and small insertion-deletions were called using both GATK and Strelka variant callers. Novel variants in the predicted off-target regions of the corrected hiPSC clones were identified as any variant within 250 bp of a predicted off-target site with a mismatch threshold of 4 that was called as reference in the parent clone by both GATK and strelka and as a variant in one of the two corrected clones by both callers (with coverage > 8 and genoqual > 25). Novel variants in the whole genome were further filtered on having no supporting reads in the parent clone, a GATK mapping quality > 50, mapping quality rank score >  − 2.5 and genotype quality > 70 in both the parent and corrected clone. Variants were annotated using GenomeComb.

### qPCR pluripotency markers hiPSC

Total RNA of five corrected hiPSC clones and the naïve parental hiPSC line was isolated using the Macherey–Nagel NucleoSpin RNA mini kit (Macherey–Nagel, 740955) according to manufacturer’s protocol. cDNA was reverse-transcribed from total RNA using the iScript cDNA Synthesis Kit (Bio-Rad Laboratories, 1708890). qPCR reactions were done in triplicate with SYBR Green Real-Time PCR master mixes (Applied Biosystems, 4309155). Expression levels were normalized to GAPDH and the ΔΔCt method was used to determine the relative levels of mRNA expression (Additional file 1: Fig. S7).

### Multiplex Amplicon Quantification

In order to determine whether the recurrent chr20q11.21 duplication [15, 16] appeared in the corrected clonal lines, we screened our corrected clones with the in-house developed Multiplex Amplicon Quantification (MAQ) technique (Agilent) consisting of a multiplex PCR amplification of fluorescently labeled target and control amplicons, followed by fragment analysis. The assay contains six target amplicons located in and around the chr20q11.21 region and five control amplicons located at randomly selected genomic positions outside the chr20q11.21 region and other known CNVs. These 11 amplicons were PCR-amplified in a single reaction containing 20 ng of genomic DNA. Peak areas of the target amplicons were normalized to those of the control amplicons. Comparison of normalized peak areas between clonal lines and references resulted in a dosage quotient (DQ) for each target amplicon, calculated by the MAQ software (MAQ-S) package (Agilent). DQ values above 1.25 were considered indicative for a duplication (Additional file 1: Fig. S6).

### Statistical analysis

Data are presented as the mean ± SD. Differences were evaluated using a *T*-test or a one-way ANOVA. Statistical analyses were performed with GraphPad Prism. A value of *P* ≤ 0.05 was considered to be significant.

## Supplementary Information

**Additional file 1: Fig. S1.** Graphical overview on protospacer-adjacent motif removal and the four possible outcome scenarios. **Fig. S2.** Integrative Genomics Viewer output file from WES data generated for the KCNQ2 R201H-P knock-in experiment in HEK293T cells. **Fig. S3.** Representative sanger sequencing files for KCNQ2 R201H-P knock-in in HEK293T using PE2max, PE4max and PE3. **Fig. S4.** Lentiviral vector and integrase-deficient lentiviral vector titering, expression in time, and edit rates. **Fig. S5.** pAIO-PE4max based removal of R201C in hiPSC over time. **Fig. S6.** Quality Control 1, Multiplex amplicon quantification for chromosome 20 duplication in hiPSC. **Fig. S7.** Quality Control 2, RT-qPCR based expression analysis of pluripotency markers in hiPSC. **Fig. S8.** Primers, pegRNAs and gBlocks used in study. **Fig. S9.** Visual representation of pegRNAs and their interacting sequences used in this study.

**Additional file 2: Table S1.** Individual data values of all data represented in this manuscript.

## Data Availability

All data generated or analyzed during this study are included in this published article, its supplementary information files and publicly available repositories. The WES dataset, NGS Miseq dataset, and ONT datasets are available via Figshare (https://doi.org/10.6084/m9.figshare.c.6490849.v2) [25]. The WGS dataset is available at EGA (EGAS00001007231). The novel pDNAs have been submitted to Addgene and are freely available for academic use (#184,444 and #184,445). Individual data values of all data represented in this manuscript can be found in Additional File 2: Table S1.

## Abbreviations

PE: Prime editing
ncRNA: Nicking RNA
dncRNA: Double nicking RNA
sncRNA: Single ncRNA
MLH1dn: MutL homolog 1
CMV: Cytomegalovirus
pAIO: All-in-one plasmid
hiPSC: Human-induced pluripotent stem cell
PAM: Protospacer-adjacent motif
Cas9n: Cas9 nickase
pegRNA: PE guide RNA
bp: Base pair
MMR: Mismatch repair
ESC: Embryonic stem cells
PEA1: All-in-one PE3 system
SNVs: Single nucleotide variants
indels: Insertions, deletions
CBh: Chicken β-actin hybrid
EF-1α: Elongation factor 1α
DEE: Developmental and epileptic encephalopathy
WES: Whole exome sequencing
PCR: Polymerase chain reaction
NGS: Next generation sequencing
HEK293T: Human embryonic kidney 293 T
LVV: Lentiviral vectors
IDLVV: Integrase-deficient lentiviral vectors
GFP: Green fluorescent protein
FACS: Fluorescent activated cell sorting
CNV: Copy number variant
MAQ: Multiplex Amplicon Quantification
WGS: Whole genome sequencing
ONT: Oxford Nanopore Technologies
