# Supplementary material for: Increased prime edit rates in KCNQ2 and SCN1A via single nicking all-in-one plasmids
Source: BMC Biol. 2023 Jul 13;21:156. doi: 10.1186/s12915-023-01646-7 (PMC10347817; doi:10.1186/s12915-023-01646-7)

**Figure S1**


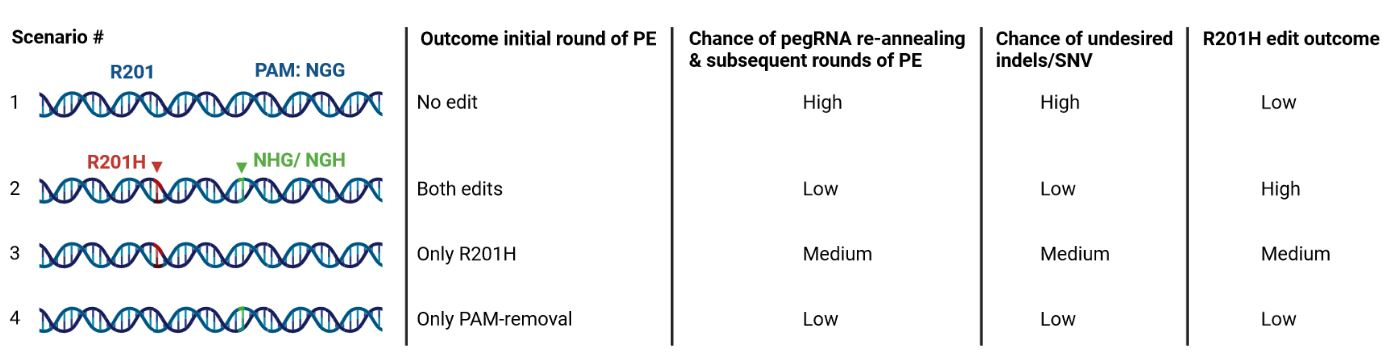


**Figure S1:** graphical overview on PAM-removal and the four possible outcome scenarios.

**Figure S2**

**
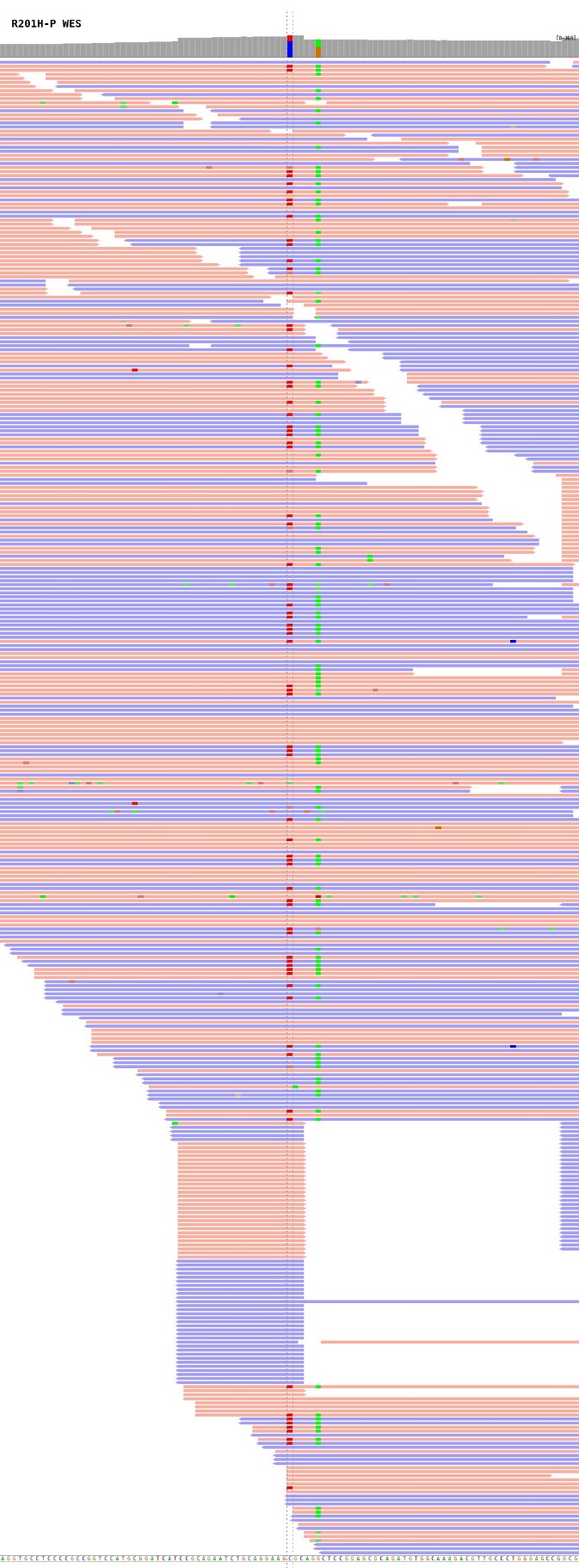
**

**Figure S2.** Visualisation of NGS (WES) data using IGV of the KCNQ2 R201H mutation knock in (red) together with an additional silent PAM-removing mutation (green) in HEK293t cells.

**Figure S3**


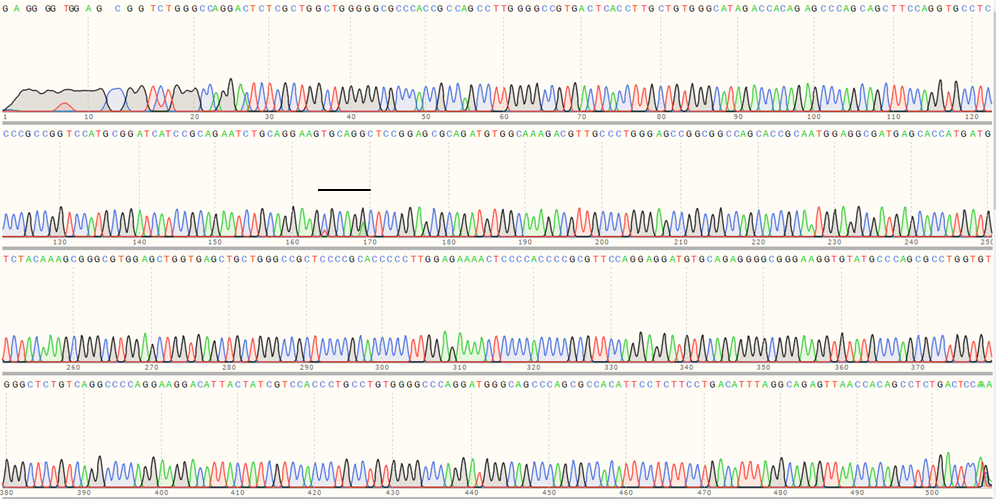


PE2max


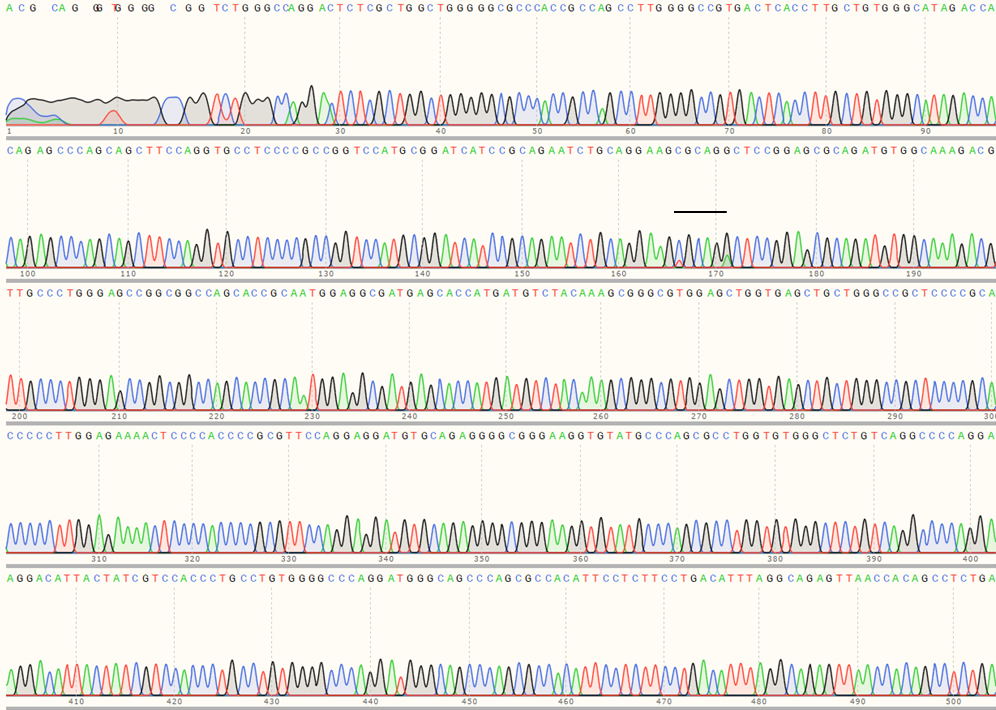


PE4max


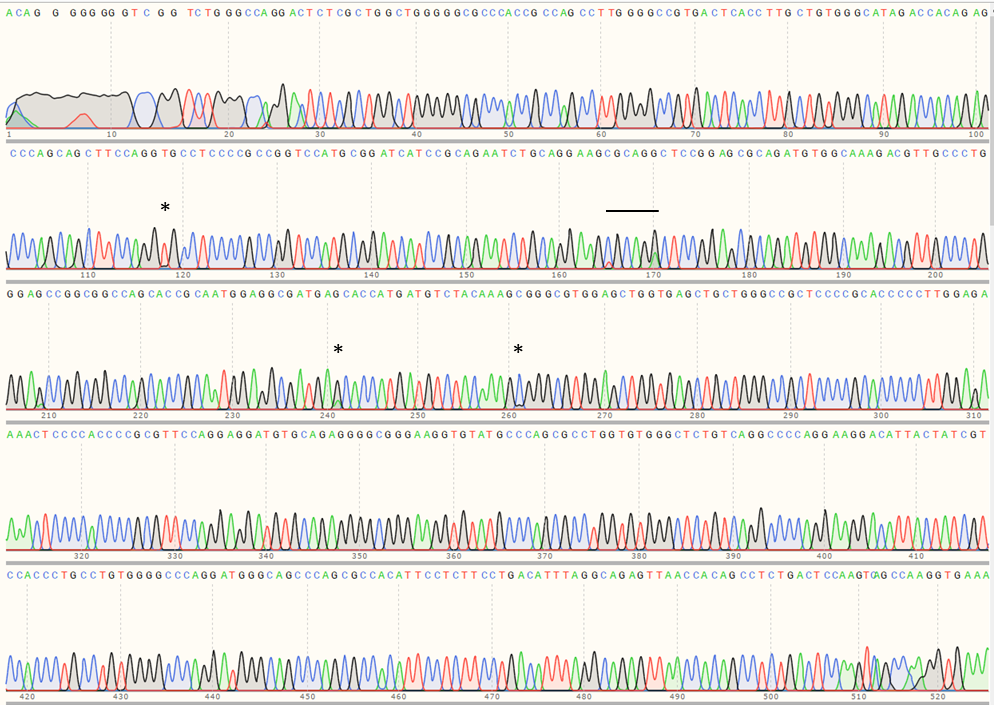


PE3

**Figure S3:** Representative sanger trace of KCNQ2 R201H knock in HEK293T using PE2max, PE4max and PE3 editing. Line indicates editing locus, asterix highlights unwanted SNVs and/or indels.

**Figure S4**


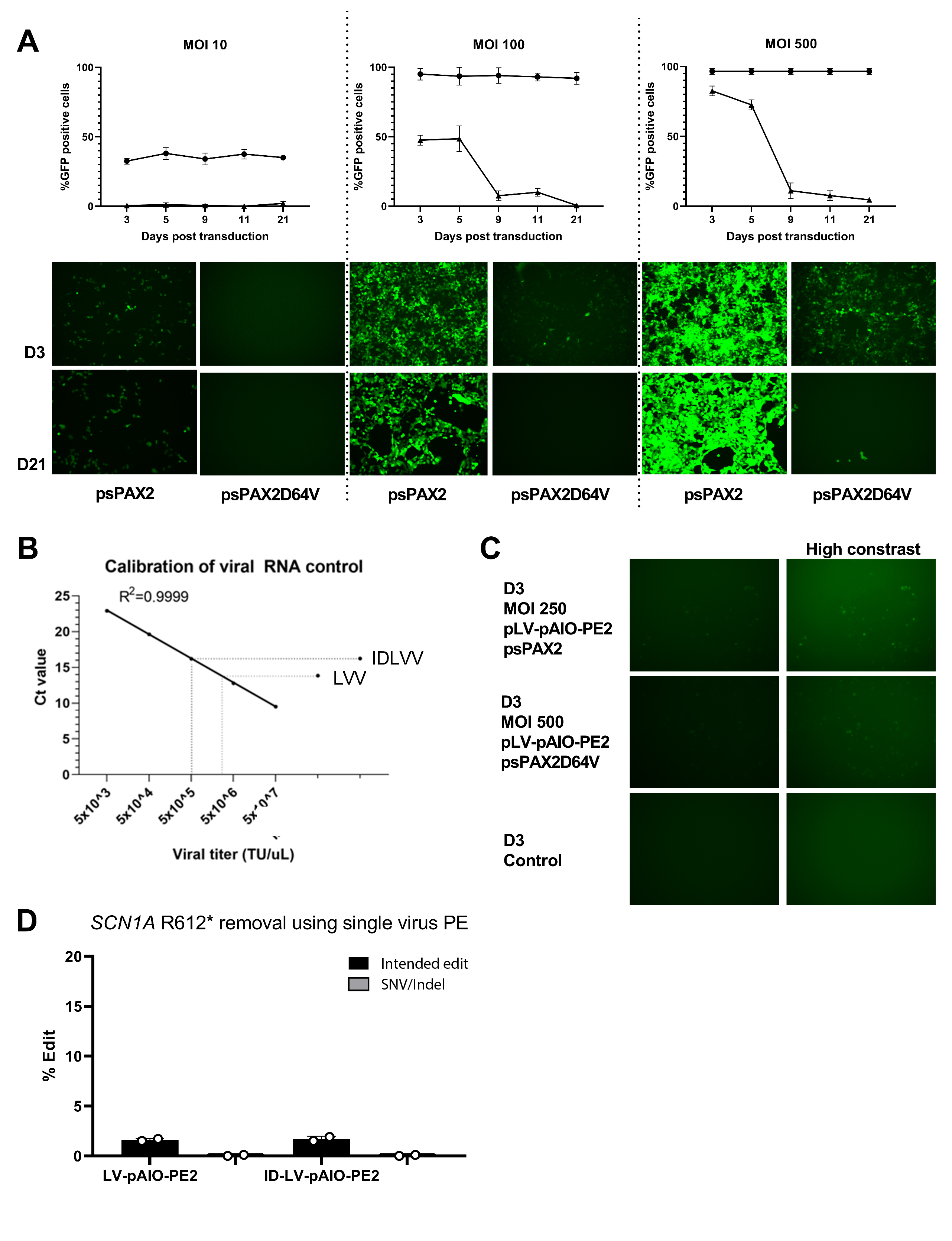


**Figure S4: (**A) GFP lentivirus produced with psPAX and psPAX-D64V packaging plasmids show stable and transient GFP expression respectively, over time with various MOI. Circles; psPAX/LV, squares; psPAX-D64V/IDLV. At D21 GFP expression is absent at 10-100 MOI of IDLV indicating transient expression in contrast to stable expression of LV over time. (B) Titering of pAIO-PE2-GFP LV and IDLV plotted on the control LV RNA calibration curve (Lenti-X-titering, Takara). (C) Imaging of pAIO-PE2-GFP LV and IDLV at MOI 250 and 500, respectively. (D) Editing rates of pAIO-PE2-GFP LV and IDLV using 250 and 500 MOI in HEK293T cells. Bar plots show the mean edit percentages ±SD, replicates are presented as individual data points (see individual data values in **Additional file 2: Table S1)**.

**Figure S5**

**
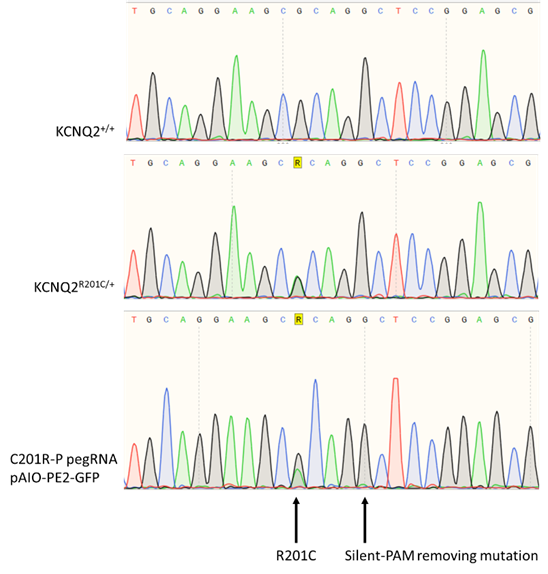
**


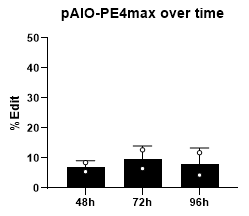


**Figure S5:** Left; hiPSC, R201C^+/-^ removal using pAIO-PE4max harvested at 48h, 72h or 96h. The different time points made little changes with regards to edit efficiency. Right: Wildtype IPSC line, KCNQ2^R201C/+^ patient-derived iPSC line and pAIO-PE2-GFP-edited iPSC line. Note the R201C mutation repair and marginal silent-PAM removing levels. Bar plots show the mean edit percentages ±SD, replicates are presented as individual data points (see individual data values in **Additional file 2: Table S1)**.

**Figure S6**


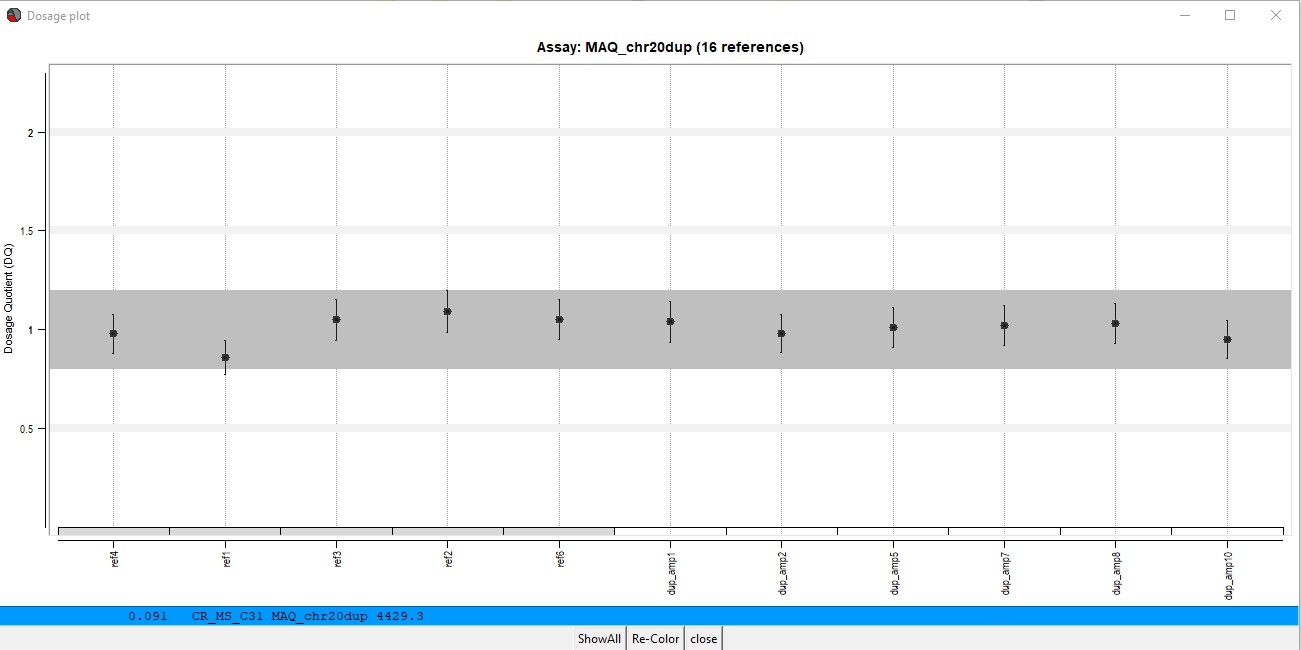


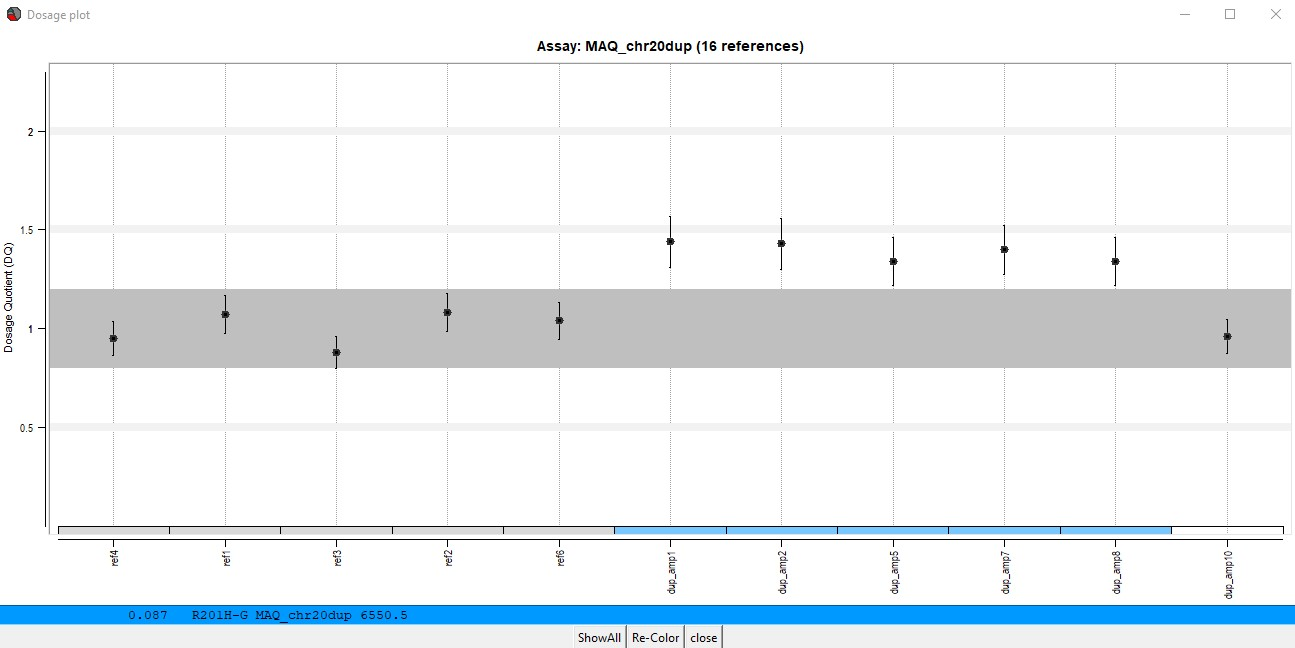


**Figure S6:** Dosage plot of the MAQ analysis. Top, example of control sample with normal copy number variation at chr20q11.21. Bottom, Example of sample carrying the chr20q11.21 duplication. Dots in the gray region (dosage quotient between 0.8 and 1.2) correspond to two copy number. The five dots between dosage quotient 1.2 to 1.5 correspond to three copy numbers. MAQ=multiplex amplicon quantification.

**Figure S7**

**Figure S7:** RT-qPCR expression analysis of pluripotency markers Oct4, Nanog and Sox2 in for five R201C corrected clonal lines (C25-C30) and the naïve parental line (R201C-N). Values were calculated using the ΔΔCt method and are normalized to GAPDH and fibroblasts expression data. Bar plots show the mean edit percentages ±SD, replicates are presented as individual data points (see individual data values in **Additional file 2: Table S1)**.

**Figure S8: Overview oligo’s**

| **PegRNAs and ncRNA oligos for U6-mCherry (5’-3’)** | | | |
| --- | --- | --- | --- |
| Design template | | | ACC-sgRNA-scaffold-3’extension-TTTTTT- (edit in red)  -NNNNNNNNNNNNNNNNNNNNNNNNNNNNNNNNN-CAA |
| KCNQ2 | pegRNA  R201H-P | fw | ACCGCAGAATCTGCAGGAAGCGCGTTTTAGAGCTAGAAATAGCAAGTTAAAATAAGGCTAGTCCGTTATCAAC  TTGAAAAAGTGGCACCGAGTCGGTGCTCCGGAGTCTGCACTTCCTGCAGATTTTTTT |
|  |  | rv | AACAAAAAAATCTGCAGGAAGTGCAGGCTCCGGAGCACCGACTCGGTGCCACTTTTTCAAGTTGATAACGGAC  TAGCCTTATTTTAACTTGCTATTTCTAGCTCTAAAACGCGCTTCCTGCAGATTCTGC |
|  | ncRNA  R201H | fw | ACCTCCATTGCGGTGCTGGCCGC |
|  |  | rv | AACGCGGCCAGCACCGCAATGGA |
| SCN1A | pegRNA  R612* | fw | ACCCGTCTCTCTCCGTGTCGTCGGTTTTAGAGCTAGAAATAGCAAGTTAAAATAAGGCTAGTCCGTTATCAAC  TTGAAAAAGTGGCACCGAGTCGGTGCGTTTGTGCCCTGACGACACGGAGATTTTTT |
|  |  | rv | AACAAAAAATCTCCGTGTCGTCAGGGCACAAACGCACCGACTCGGTGCCACTTTTTCAAGTTGATAACGGACTA  GCCTTATTTTAACTTGCTATTTCTAGCTCTAAAACCGACGACACGGAGAGAGACG |
|  | pegRNA  *612R | fw | ACCGCGTCTCTCTCCGTGTCGTCGTTTTAGAGCTAGAAATAGCAAGTTAAAATAAGGCTAGTCCGTTATCAACT  TGAAAAAGTGGCACCGAGTCGGTGCTTTGTGCCCCGACGACACGGAGAGTTTTTT |
|  |  | rv | AACAAAAAACTCTCCGTGTCGTCGGGGCACAAAGCACCGACTCGGTGCCACTTTTTCAAGTTGATAACGGACTA  GCCTTATTTTAACTTGCTATTTCTAGCTCTAAAACGACGACACGGAGAGAGACGC |
|  | pegRNA  L622V | fw | ACCATGACCTACTGGTCTGACTCGTTTTAGAGCTAGAAATAGCAAGTTAAAATAAGGCTAGTCCGTTATCAAC  TTGAAAAAGTGGCACCGAGTCGGTGCACAGCAACGTGAGTCAGACCAGTAGGTTTTTTT |
|  |  | rv | AACAAAAAAACCTACTGGTCTGACTCACGTTGCTGTGCACCGACTCGGTGCCACTTTTTCAAGTTGATAACGGA  CTAGCCTTATTTTAACTTGCTATTTCTAGCTCTAAAACGAGTCAGACCAGTAGGTCAT |
|  | pegRNA  L622M | fw | ACCATGACCTACTGGTCTGACTCGTTTTAGAGCTAGAAATAGCAAGTTAAAATAAGGCTAGTCCGTTATCAACT  TGAAAAAGTGGCACCGAGTCGGTGCACAGCAACATGAGTCAGACCAGTAGGTTTTTTT |
|  |  | rv | AACAAAAAAACCTACTGGTCTGACTCATGTTGCTGTGCACCGACTCGGTGCCACTTTTTCAAGTTGATAACGGA  CTAGCCTTATTTTAACTTGCTATTTCTAGCTCTAAAACGAGTCAGACCAGTAGGTCAT |
|  | ncRNA  612-623 | fw | ACCTGATGAGCACAGCACCTTTG |
|  |  | rv | AACCAAAGGTGCTGTGCTCATCA |
| EMX1 | pegRNA  K263N | fw | ACCGAGTCCGAGCAGAAGAAGAAGTTTTAGAGCTAGAAATAGCAAGTTAAAATAAGGCTAGTCCGTTATCAACT  TGAAAAAGTGGCACCGAGTCGGTGCGTGATGGGAGCCCTTGTTCTTCTGCTCGGTTTTTT |
|  |  | rv | AACAAAAAACCGAGCAGAAGAACAAGGGCTCCCATCACGCACCGACTCGGTGCCACTTTTTCAAGTTGATAACG  GACTAGCCTTATTTTAACTTGCTATTTCTAGCTCTAAAACTTCTTCTTCTGCTCGGACTC |
|  | pegPAM  G265C | fw | ACCGAGTCCGAGCAGAAGAAGAAGTTTTAGAGCTAGAAATAGCAAGTTAAAATAAGGCTAGTCCGTTATCAACT  TGAAAAAGTGGCACCGAGTCGGTGCATGGGAGCACTTCTTCTTCTGCTCGGTTTTTTT |
|  |  | rv | AACAAAAAAACCGAGCAGAAGAAGAAGTGCTCCCATGCACCGACTCGGTGCCACTTTTTCA  AGTTGATAACGGACTAGCCTTATTTTAACTTGCTATTTCTAGCTCTAAAACTTCTTCTTCTGCTCGGACTCGGT |
|  | ncRNA  263-265 | fw | ACCGACATCGATGTCCTCCCCAT |
|  |  | rv | AACATGGGGAGGACATCGATGTC |

| **Oligos for pAIO cloning (5’-3’)** | |
| --- | --- |
| pLV-PE2_fw | GATGGTCCGATTTCAGCAGGTGATGAGGTTAGCAGTCTCACCTGCGGGCAGATCGAACCTGCAGGTTCCACTGGGACGATTACATGCATCTGGAT |
| pLV-PE2 rv | CGATCCAGATGCATGTAATCGTCCCAGTGGAACCTGCAGGTTCGATCTGCCCGCAGGTGAGACTGCTAACCTCATCACCTGCTGAAATCGGACCAT |
| pLV-AIO-PE4max_fw | CCGGTTCAGACGCGT |
| pLV-AIO-PE4max_rv | CCGGACGCGTCTGAA |

| **gBlock pegRNA inserts for pAIO-PE2/pAIO-PE4max (5’-3’)** | |
| --- | --- |
| Design template: | CGGATCGATTTCAGCAGGTGCCATGTGAGGGCCTATTTCCCATGATTCCTTCATATTTGC  ATATACGATACAAGGCTGTTAGAGAGATAATTGGAATTAATTTGACTGTAAACACAAAGAT  ATTAGTACAAAATACGTGACGTAGAAAGTAATAATTTCTTGGGTAGTTTGCAGTTTTAAAA  TTATGTTTTAAAATGGACTATCATATGCTTACCGTAACTTGAAAGTATTTCGATTTCTTGGC  TTTATATATCTTGTGGAAGGACGAAACACC  -sgRNA-scaffold-3’extension-TTTTTT-AGAGCCACCTGCGGGCAGATTAGGC |
| *612R | GCGTCTCTCTCCGTGTCGTCGTTTTAGAGCTAGAAATAGCAAGTTAAAATAAGGCTAGTCC  GTTATCAACTTGAAAAAGTGGCACCGAGTCGGTGCTTTGTGCCCCGACGACACGGAGAG |
| C201R | GCAGAATCTGCAGGAAGCACGTTTTAGAGCTAGAAATAGCAAGTTAAAATAAGGCTAGTCC  GTTATCAACTTGAAAAAGTGGCACCGAGTCGGTGCTCCGGAGCCTGCGCTTCCTGCAGATT |
| C201R-P | GCAGAATCTGCAGGAAGCACGTTTTAGAGCTAGAAATAGCAAGTTAAAATAAGGCTAGTCC  GTTATCAACTTGAAAAAGTGGCACCGAGTCGGTGCTCCGGAGTCTGCGCTTCCTGCAGATT |

| **Primers (5’-3’)** | |
| --- | --- |
| **PCR primers** | |
| KCNQ2 forward | TTCACCTGCTGACTTGGAGT |
| KCNQ2 reverse | ACCCAGGGCTCTTGAAGCAAA |
| SCN1A forward | CGTGGCTCCCTATTTTCACC |
| SCN1A reverse | CCACCAACCAAGGAAACCAC |
| EMX1 forward | TGCCATCCCCTTCTGTGAAT |
| EMX1 reverse | CTTGTCCCTCTGTCAATGGC |
| KCNQ2 ONT amplicon Forward  Black: primer ; red: Tag | TTTCTGTTGGTGCTGATATTGCGTCAGGAAGAGGAATGTGGC |
| KCNQ2 ONT amplicon Reverse  Black: primer ; red: Tag | ACTTGCCTGTCGCTCTATCTTCGTTCGCGGCAGGAAGGTG |
| **Additional Sequencing primers** | |
| U6 forward | GAGGGCCTATTTCCCATGATT |
| M13 reverse | GTCATAGCTGTTTCCTG |
| Cas9 forward | CAGTACGCCGACCTGTTTCT |
| pAIO forward | GTGCAGGGGAAAGAATAGTAGACA |
| pAIO reverse | TAGAACCCAGAGATCGCT |
| EGFP forward | GACGTAAACGGCCACAAGTT |
| EGFP reverse | GGGCATCGACTTCAAGGAGG |
| CMV forward | CGCAAATGGGCGGTAGGCGTG |

**Figure S9. Visual presentation of pegRNA used in this study.**

**EMX1: PegRNA-K263N**


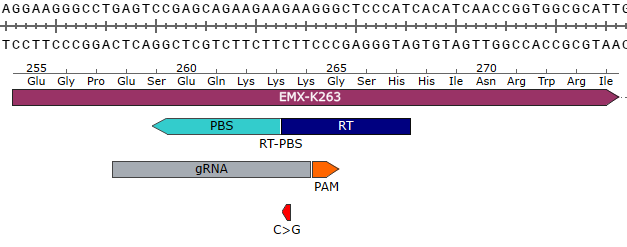


**EMX1: PegRNA-G265C**


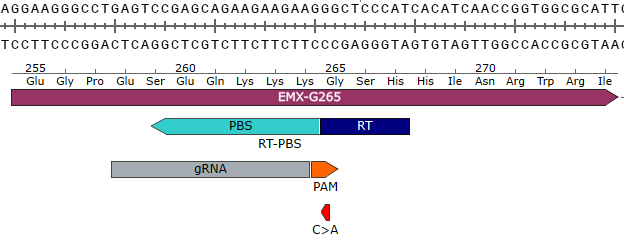


**SCN1A: PegRNA-R612***


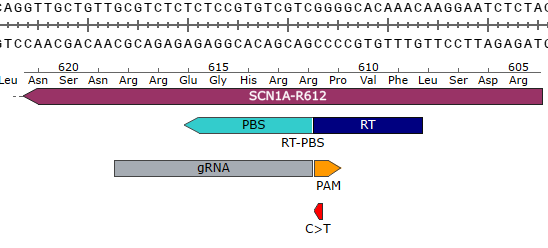


**SCN1A: PegRNA-*612R**


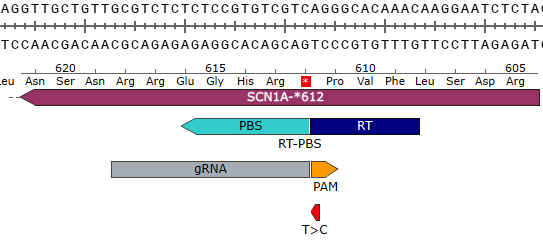


**KCNQ2: PegRNA-R201H-P**


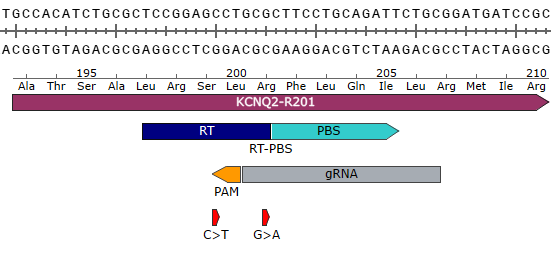


**SCN1A: PegRNA-L622V**


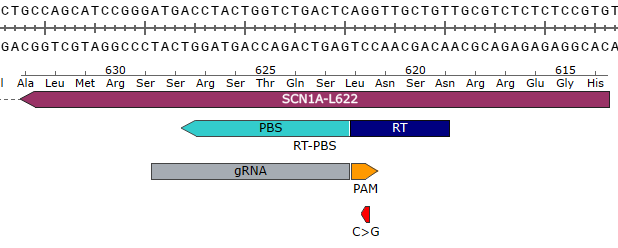


**SCN1A: PegRNA-L622M**


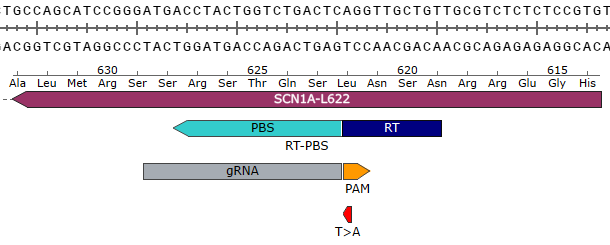


**KCNQ2: PegRNA-C201R**


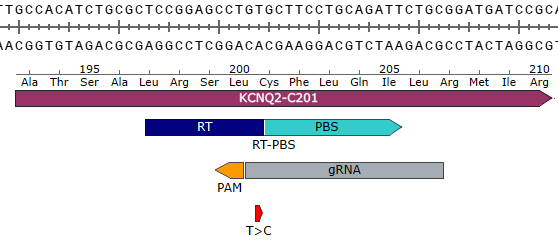


**KCNQ2: PegRNA-C201R-P**


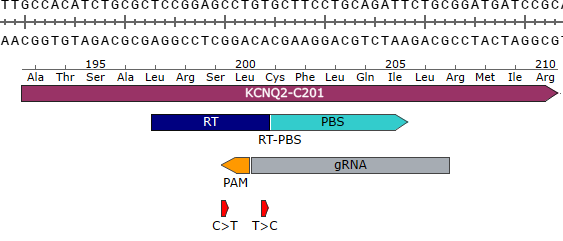

Supplement: Supplementary file 1 — Additional file 1: Fig. S1. Graphical overview on protospacer-adjacent motif removal and the four possible outcome scenarios. Fig. S2. Integrative Genomics Viewer output file from WES data generated for the KCNQ2 R201H-P knock-in experiment in HEK293T cells. Fig. S3. Representative sanger sequencing files for KCNQ2 R201H-P knock-in in HEK293T using PE2max, PE4max and PE3. Fig. S4. Lentiviral vector and integrase-deficient lentiviral vector titering, expression in time, and edit rates. Fig. S5. pAIO-PE4max based removal of R201C in hiPSC over time. Fig. S6. Quality Control 1, Multiplex amplicon quantification for chromosome 20 duplication in hiPSC. Fig. S7. Quality Control 2, RT-qPCR based expression analysis of pluripotency markers in hiPSC. Fig. S8. Primers, pegRNAs and gBlocks used in study. Fig. S9. Visual representation of pegRNAs and their interacting sequences used in this study. [file 12915_2023_1646_MOESM1_ESM.docx]
